# A trigonal coordination of Au(I) phosphane complexes stabilized by O–H^**⋯**^X (X = Cl^–^, Br^–^, I^–^) interactions

**DOI:** 10.1007/s00706-021-02843-2

**Published:** 2021-09-15

**Authors:** Petra Gründlinger, Cezarina Cela Mardare, Thorsten Wagner, Uwe Monkowius

**Affiliations:** 1 Institute of Experimental Physics–Surface Science Division, Johannes Kepler University Linz, Altenberger Straße 69, 4040 Linz, Austria; 2 Institute of Chemical Technology of Inorganic Materials, Johannes Kepler University Linz, Altenberger Straße 69, 4040 Linz, Austria; 3 Faculty of Medicine/Dental Medicine, Department of Physics and Chemistry of Materials, Danube Private University, Steiner Landstraße 124, 3500 Krems an der Donau, Austria; 4 School of Education, Chemistry, Johannes Kepler University Linz, Altenberger Straße 69, 4040 Linz, Austria

**Keywords:** Gold complexes, Crystal structure, Tri-coordinate gold(I), Hydrogen bonds

## Abstract

In this work, we show that intramolecular hydrogen bonding can be used to stabilize tri-coordinated phosphane-gold(I) complexes. Two molecular structures of 2-(diphenylphosphino)benzoic acid (L) coordinated to a gold(I) atom were determined by single-crystal X-ray diffraction. The linear L–Au–Br shows a standard linear coordination and dimerizes via hydrogen bonds of the carboxylic acid. Upon addition of two additional phosphane ligands the complex [L_3_Au]X is formed which is stabilized by three intramolecular –C(O)O–H^**…**^X hydrogen bonds as proven by the X-ray structure of the respective chlorido-complex. X-ray powder diffractograms suggest the same structure also for X^–^ = Br^–^ and I^–^.

**Graphic abstract:**

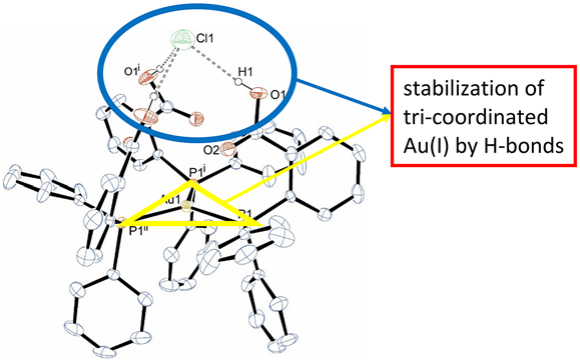

**Supplementary Information:**

The online version contains supplementary material available at 10.1007/s00706-021-02843-2.

## Introduction

The structural chemistry of gold(I) complexes is dominated by a linear coordination, although higher coordination numbers are not particularly rare [1–4]. Interestingly, for isoelectronic cations like Ag(I), Pt(0), or Hg(II) the preference for a linear coordination is much weaker and they usually form complexes with coordination numbers higher than two. Due to a possible formation of aurophilic interactions a huge structural diversity is observed for various types of ligands in their gold(I) complexes. Especially well investigated are phosphane complexes of gold(I) for a variety of reasons: (i) they are easy to prepare, (ii) a wide variety of structural diverse phosphanes are commercial available, (iii) they are relatively stable under ambient condition, although sometimes light sensitive, (iv) with ^31^P NMR spectroscopy, a very convenient and sensitive method for a fast and efficient characterisation is available, (v) gold(I) phosphane complexes have been proven to be very useful in many different applications and can be found as luminescent material, catalyst, or pharmaceutical active agent [5–14].

However, there are surprising few systematic structural studies on the extension of the coordination sphere of mononuclear gold(I) complexes beyond the linear geometry. By adding additional phosphane ligands R_3_P to complexes of the type R_3_P–Au–X (X = mostly anionic ligand, e.g. halides or pseudohalides), different coordination environments are conceivable and have been reported. For a P:Au ration of 2:1, either the distorted trigonal-planar and neutral form (R_3_P)_2_AuX or the ionic form [(R_3_P)_2_Au]X are known. In the latter case, the gold atom is often in a linear, sometimes more or less distorted environment with the halide non- or weakly bonded [15–22]. The neutral form is often found for rigid bidentate phosphanes like 1,2-bis(diphenylphosphino)carborane or 1,2-bis(diphenylphosphino)benzene and have the form (P^P)AuCl [23–27].

The ratios 3:1 and 4:1 is often but not exclusively found for X = weakly coordinating anions and these compounds contain a trigonal-planar [(R_3_P)_3_Au]^+^ or tetrahedral [(R_3_P)_4_Au]^+^ coordinated cation, respectively [12, 28–30]. The latter is also the highest coordination number found for phosphane-gold(I) complexes but very rare for monodentate phosphanes [31–33]. Seldom, neutral tetrahedral species are found (R_3_P)_3_AuX (X = halide) [34–37]. Again, rigid bidentate phosphane ligands facilitate higher coordination numbers. In these cases, several complex cations of the form [(P^P)AuL]^+^ (L = phosphane or carbene ligand) and [(P^P)_2_Au]^+^ have been reported [38–43].

Mononuclear, tri-coordinated gold(I) cations are interesting as they often feature phosphorescence based on mainly ligand field excited states [44–46]. Due to Jahn–Teller distortion, these complexes feature T-shape geometries in the excited state [47]. This strong distortion is also the reason for the huge Stokes shift, broad and unstructured emission band and the fact that luminescence is often relatively weak in solution or only observed at cryogenic temperature [48]. It should be mentioned that there are also several examples of bidentate phosphane ligands which form dinuclear gold(I) complexes with short Au^**…**^Au distances. However, such phosphorescence is based on aurophilic interactions and also observable at room temperature [49].

For monodentate phosphanes steric and electronic properties seem to subtly influence the stability of gold(I) complexes with higher coordination numbers [50]. In the literature, the complete series of (Ph_3_P)_n_AuX complexes (with *n* = 1–3, X = anion) is described whereas there are no reports on the existence of other, even very similar phosphanes which would form the whole set of possible complexes [45, 51]. Taking the dynamic behaviour of gold(I) complexes in solution into account, the polarity of the solvent might also play a crucial role as the formation of ionic or neutral complexes are sometimes determined by the polarity of the solvent [52, 53]. Besides sterics, it had been reasoned that higher coordination numbers could be established with (moderate) electron-poor ligands and indeed several gold(I) complexes bearing such phosphane ligands have been reported [29, 31, 36, 37]. This observation is conform with the fact that the less electron-donating Ph_3_E (E = As, Sb) readily form the respective gold complexes [(Ph_3_E)_4_Au]BF_4_ [54]. However, there are also examples of electron-donating phosphanes forming highly coordinated gold(I) atoms [28]. In this contribution, we would like to introduce a concept to stabilize mononuclear, tri-coordinate gold(I) complexes via E–H^**…**^X (E = O, N) interactions. A previously reported example is a complex, where an amide N–H functions as hydrogen-bond donor to the coordinated chloride facilitating tri-coordination of the form (Ph_2_RP)_2_AuCl (R = *o*-trifluoroacetanilide) [55]. An extensive hydrogen-bond network was also found in the tetrahedral gold(I) complex bearing 1,3,5-triaza-7-phosphaadamantane [31]. Similar, the tri-coordinated gold(I) complex bearing the trisulfonated triphenylphosphane is stabilized via an excessive network between the sulfonate groups and Cs^+^ counterions [29].

## Results and discussion

### Synthesis

**Fig. 1:**
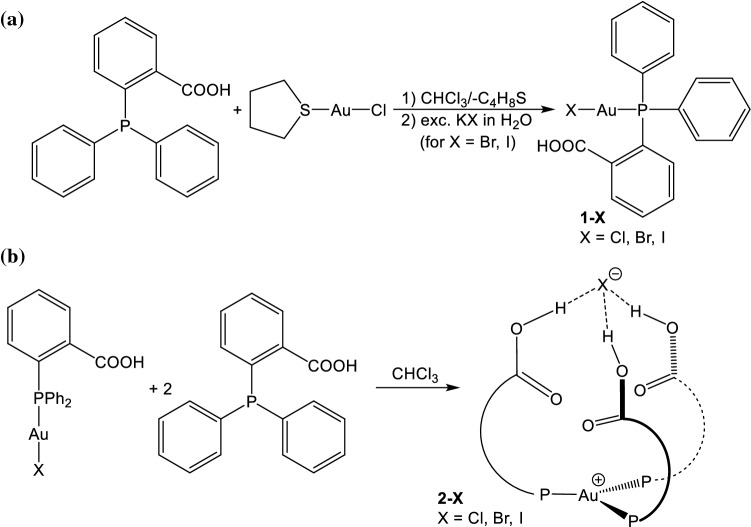
Reaction scheme for the synthesis of mono (**a**) and trifold (**b**) coordinate Au(I) complexes bearing the diphenylphosphino-benzoic acid ligand (X = Cl, Br, I)

Reaction of 2-(diphenylphosphino)benzoic acid, Ph_2_(*o*-BzOH)P, with equimolar amounts of (tht)AuCl (tht = tetrahydrothiophene) leads to the formation of 2-(diphenylphosphino)benzoic acid gold(I) chloride, Ph_2_(*o*-BzOH)PAuCl, **1-Cl**. It should be mentioned that various gold(I) complexes bearing a diphenylphosphino-benzoic acid—both *ortho* and *para*—have been reported, among them also **1-Cl** [56–59]. Through metathesis with KBr and KI the bromido and iodido congener **1-Br** and **1-I** are easily accessible. Upon addition of two further equivalents of the phosphane ligand, [Au(PPh_2_(*o*-BzOH))_3_]X (X = Cl, Br, I), **2-X**, is formed (Fig. 1).

### NMR spectroscopy

Due to the low solubility of the tri-coordinate gold(I) complexes **2-X**, a thorough characterization by NMR spectroscopy was only possible for **1-X**. Nevertheless, we conducted ^31^P{^1^H} NMR studies to get some basic information by adding different equivalents of the phosphane ligand to solutions of **1-X** in CDCl_3_ (see Supporting Information, Fig. S1). The ^31^P NMR shift of the free ligand is at − 4.1 ppm. Upon adding (tht)AuCl in equimolar amounts, the peak is shifted to 36.1 ppm for **1-Cl**. For **1-Br** and **1-I** the peaks can be found at 38.3 and 42.5 ppm, respectively. An additional equivalent of the phosphane ligand gives a very broad signals at ~ 45 ppm, the respective peaks of for the bromide (~ 42.5 ppm) and iodide (~ 39 ppm) are slightly sharper. These observations are indicative for a dynamic behaviour of the present gold(I) species, presumably due to an equilibrium between the neutral tri- and ionic di-coordinated form according to [AuL_2_X] ⇄ [AuL_2_]^+^+ X^–^ [L = PPh_2_(*o*-BzOH)]. After the addition of the third equivalent of the ligand, the ^31^P NMR signals either almost vanish (for **2-Br** and **2-I**, both at ~ 41 ppm) or have a low intensity (**2-Cl**, ~ 43 ppm) reflecting the low solubility of the complexes. Indeed, already with the addition of the second equivalent, some white precipitate forms. These observations are indicative for a lower solubility of the **2-Br**/**2-I** compared to **2-Cl**, which might be the reason for the unsuccessful attempts of growing single crystals of all prepared complexes (vide infra). Interestingly, upon addition of an excess of the ligand to **2-Cl**, not only the peak of the complex, but also the free ligands are broad possible due to an exchange between bound and free phosphane ligands.

### Structural studies

**Fig. 2:**
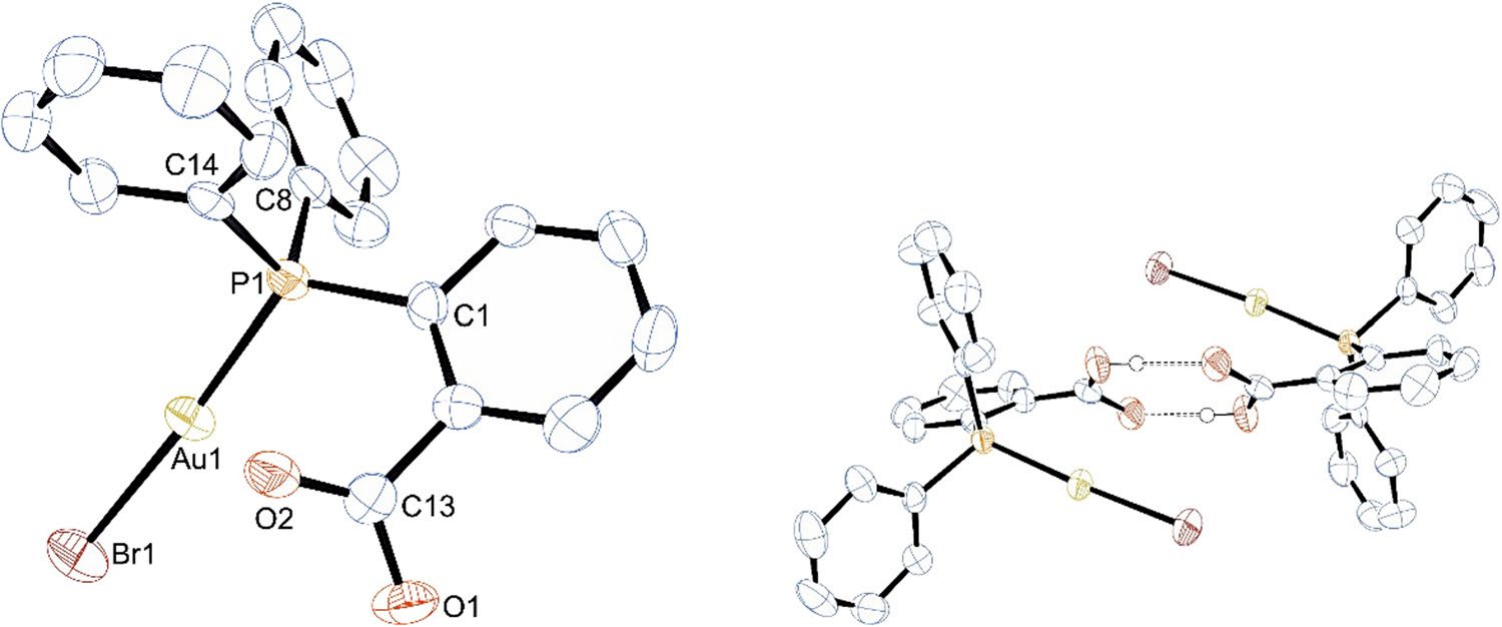
Left: molecular structure of 2-(diphenylphosphino)benzoic acid gold(I) bromide, **1-Br** (H omitted for clarity); right: dimerization of **1-Br** in the solid state via hydrogen bonds. Selected bond lengths in Å and angles in °: Au1–Br1 2.3957(4), Au1–P1 2.247(1), P1–C1 1.825(3), P1–C8 1.817(3), P1–C14 1.815(3), Br1–Au1–P1 175.38(2), Au1–P1–C1 119.8(1), Au1–P1–C8 108.7(1), Au1–P1–C14 110.9(1), O1–C13–O2 124.7(3)

Crystals suitable for single-crystal X-ray diffraction analysis of 2-(diphenylphosphino)benzoic acid gold(I) bromide, **1-Br**, could be obtained by slow gas diffusion of *n*-pentane into a concentrated solution of **1-Br** in DCM (Fig. 2). The compound crystallizes in the monoclinic space group *P*2_1_/*n*. Crystals of chlorido-complex contains one molecule of CHCl_3_ [59], hence **1-Cl** and **1-Br** are not isostructural. However, the arrangement of the molecules is very similar in both crystals: in their crystals the complexes dimerize via hydrogen bonds between the carboxylic groups (Fig. 2). The O⋯O distance of two adjacent gold complexes is 2.686(3) Å, which is typical for O–H⋯O of hydrogen bonded carboxylic acid groups [60, 61]. The gold atom is linearly coordinated with an angle Br1–Au1–P1 of 175.38(2)°. The distance Au1–P1 is 2.247(1) Å and only slightly shorter than that of the chlorido congener {2.26(1) Å [59]}. The Au1–Br1 bond length is 2.3957(4) Å and typical for R_3_P–Au–Br complexes [62, 63]. Contrary to the *para*-substituted homologue, there are no aurophilic interactions present [64]. The shortest Au–Au distance of 7.431(1) Å is far beyond the aurophilicity limit of ~ 3.5 Å [65].

In the course of the synthetic work, also a few crystals of the complex [Au(PPh_2_(*o*-BzOH))_2_I] could be isolated, however, we were not able to synthesis this compound in pure form. This is somewhat surprising as the homologue chlorido-complex is known [56]. Unfortunately, the crystals contain solvent molecules which could not be modelled satisfactorily and thus lead to poor structural refinement data. For the sake of completeness, we have added this structure to the supporting information but will not further discuss it here (Fig. S2 + Table S1).

**Fig. 3:**
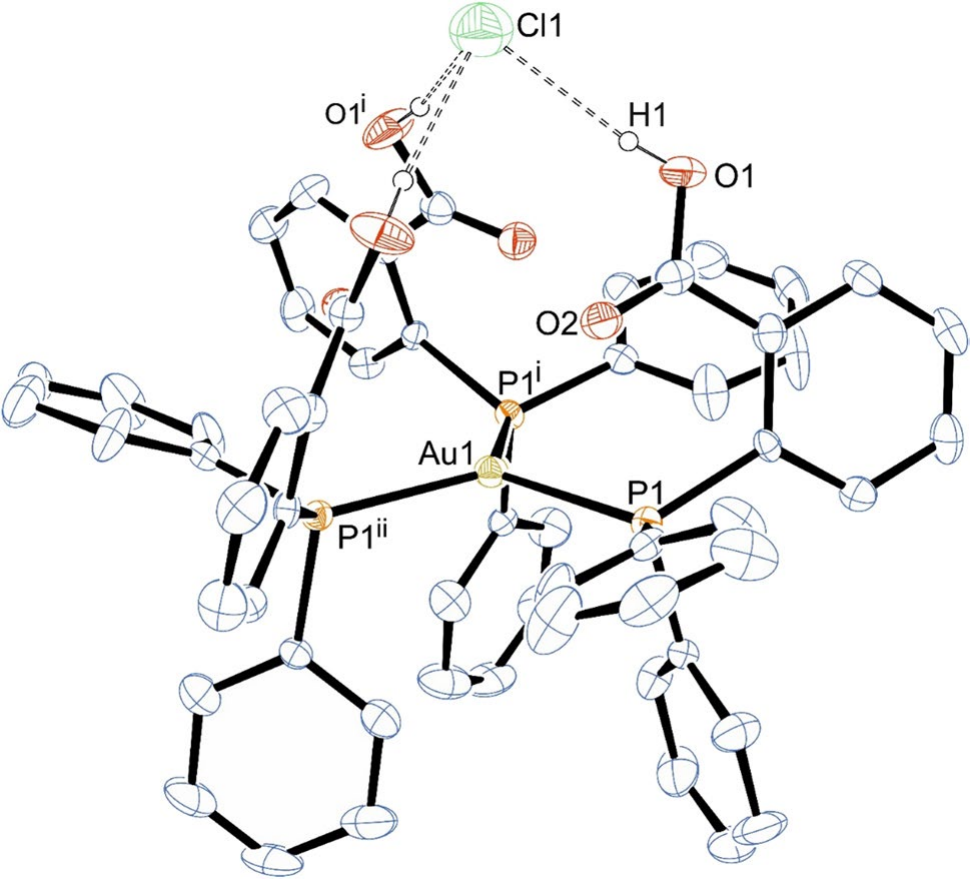
Molecular structure of **2-Cl** (with exception of carboxylic acid all other H atoms omitted for clarity). Selected bond lengths in Å and angles in °: Au1–P1, P1–C7 1.81(1), O1–H1 0.821, O^**…**^Cl 3.108, P1–Au1–P1^i^ 119.57(1)

Complex **2-Cl** crystallizes in the cubic space group *Pa*$\stackrel{\mathrm{-}}{3}$. The asymmetric unit consist of one third of the molecule. The gold atom is coordinated by three phosphorus atoms of the phosphane ligands in trigonal-planar arrangement with a *C*_3_-axis passing through the gold and chlorine atoms. Therefore, there is only one Au1–P1 distance with 2.412(2) Å, which is considerably longer than those of **1-Br**. The angle P1–Au1–P1 is 119.57(1)° and sums up to 358.71°. The carboxylic acid groups are oriented towards the *C*_3_-axis. This arrangement leads to intense hydrogen bonding of the carboxylic acid proton towards the chloride anion locking it in their center (Fig. 3). The O–Cl distance of the O–H^**…**^Cl group is 3.108 Å indicative for moderate hydrogen bonds [60, 61]. The previously reported complex of the form (Ph_2_RP)_2_AuCl (with R = *o*-trifluoroacetanilide) feature comparable N–H^**…**^Cl hydrogen bonds (N–Cl ~ 3.2 Å), which stabilize the trigonal-planar coordination environment around the Au(I) atom [55]. Thus, the utilization of hydrogen bonds might be regarded as a general synthetic strategy to stabilize otherwise not isolable, tri-coordinated Au(I) complexes.

**Fig. 4:**
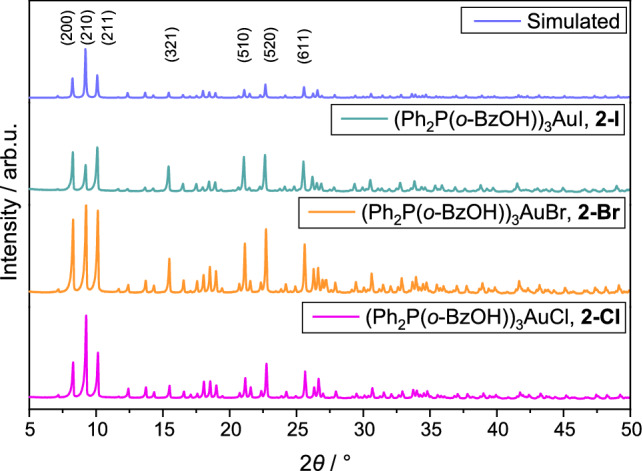
PXRD of **2-Cl**, **2-Br** and **2-I**. Top: simulated pattern of **2-Cl**

Unfortunately, single crystals suitable for structural analysis could only be obtained for the chlorido compound. Although numerous attempts to grow crystals for the bromido and iodido congeners were undertaken, only powders could be isolated. The reason is their general very low solubility in common organic solvents. Immediately after addition of the third equivalent of the phosphane ligand, the complexes precipitate out of solution. Various attempts with different solvents, concentrations, and crystallization conditions always lead to micro-crystalline samples. Hence, powder X-ray diffractograms were recorded for comparing the samples and ascertain whether they crystalize isostructural. The single-crystal structure data of **2-Cl** are used to simulate its powder pattern. As depicted in Fig. 4, the diffraction patterns are similar for all three compounds and also match the simulated pattern of **2-Cl**. The most intense peaks are generated from the (200), (210), and (211) planes. Another characteristic pattern arises from the (510) and (520) planes. Therefore, it can be inferred that the bromido and iodido complexes are isostructural to the chlorido congener featuring a tri-coordinated Au(I) atom.

Although tri-coordinate phosphane-gold(I) complexes often feature photo-luminescence, we could not detect any light emission upon excitation with UV-light.

## Conclusion

In this work, we suggest a new concept to stabilize coordination numbers larger than two of gold(I) in its phosphane complexes beyond the dominating standard linear coordination. Using intramolecular hydrogen bonds, it is possible to stabilize a complex of the form [L_3_Au]X (L = 2-(diphenylphosphino)benzoic acid, X = Cl, Br, I). The molecular structure of [L_3_Au]Cl could be determined by single-crystal X-ray diffraction, whereas those of X = Br and I are deduced by a similar pattern of powder-diffractograms. We suggest intramolecular hydrogen bonds for facilitating tri-coordination of sometimes difficult to stabilize tri-coordinated gold(I) atoms. Further synthetic work has to prove the scope of this concept, particularly for complexes which are better soluble than **2-X**. Formation of **2-X** is clearly favoured by the low solubility of these complexes whereas in solution, both coordinated ligands as well as intramolecular hydrogen bonds would have to compete with possibly interfering solvent molecules.

### Experimental

All commercially available solvents and starting materials were used without further purification. 2-(Diphenylphosphino)benzoic acid was purchased from abcr. (tht)AuCl (tht = tetrahydrothiophene) was prepared according to a reported procedure [66].

NMR spectra were recorded on a Bruker 300 MHz Avance III spectrometer. For all measurements, deuterated solvents were used. Chemical shifts are related to the residual solvent signal and are stated to the *δ*-convention in ppm. HR-MS measurements were carried out on an Agilent 6520 QTOF mass spectrometer with an ESI source. X-ray powder diffraction was recorded on a Philips X’pert Pro diffractometer operated in Bragg–Brentano geometry and employing CuK_α_ radiation (*λ* = 1.541874 Å). The patterns were acquired using a step size of 0.008° with a counting time of 60 s per step; the samples were rotated with 15 rotations per min.

Single-crystal structure analysis was carried out at room temperature on a Bruker D8 Quest ECO diffractometer with graphite-monochromated MoK_α_ radiation (*λ* = 0.71073 Å). The structures were solved by direct methods (SHELXS-97 [67]) and refined by full-matrix least-squares on *F*^2^ {SHELXL-2014/7 [68]}. The H atoms were calculated geometrically, and a riding model was applied in the refinement process. Crystallographic details can be found in Table 1 and S1. CCDC 2094495 (**1-Br**), 2094497 (**2-Cl**), 2094497 ([Au(PPh_2_(*o*-BzOH))_2_I]), contain supplementary crystallographic data for this paper. This information can be obtained free of charge via https://www.ccdc.cam.ac.uk/structures/

**Table 1:** Crystal data and data collection and structure refinement details for **1-Br** and **2-Cl**

	**1-Br**	**2-Cl**
Empirical formula	C_19_H_15_AuBrO_2_P	C_57_H_45_AuClO_6_P_3_
*M*_*r*_/g mol^−1^	583.16	1151.26
Crystal size/mm^3^	0.29 × 0.10 × 0.05	0.46 × 0.34 × 0.08
Crystal system	monoclinic	cubic
Space group	*P*2_1_/*n*	*Pa*$\stackrel{\mathrm{-}}{3}$
*a*/Å	15.3001(10)	21.4601(4)
*b*/Å	7.4332(4)	21.4601(4)
*c*/Å	17.4332(10)	21.4601(4)
*α*/°	90	90
*β*/°	114.829(1)	90
*γ*/°	90	90
*V*/Å^3^	1800.60(18)	9883.1(6)
*ρ*_calcd._/g cm^3^	2.151	1.547
*Z*	4	8
*μ*(MoK_α_)/mm^−1^	10.49	3.18
*T*/K	296	296
*Θ* Range/°	2.9–26.3	2.7–23.4
Reflections collected	57,743	67,167
Unique reflections	3644	2409
Observed reflections I > 2σ(I)	3181	1447
Absorption correction	Multi-scan	Multi-scan
*T*_min_/*T*_max_	0.15/0.60	0.32/0.78
∆*ρ*_fin_(max/min)/e Å^−3^	0.46, − 0.49	1.31, − 0.69
*R*1 [I ≥ 2σ(I)]	0.020	0.048
*wR*2	0.042	0.131
CCDC	2094495	2094497

#### **Chlorido-[2-(diphenylphosphino)benzoic acid]gold(I), (1-Cl, C**_**19**_**H**_**15**_**AuClO**_**2**_**P) 1-Cl**

 was prepared according to a modified literature method [56, 57]: (tht)AuCl (98.8 mg, 0.308 mmol) was dissolved in 20 cm^3^ DCM and 2-(diphenylphosphino)benzoic acid (91 mg, 0.30 mmol) was added and the mixture was stirred for 2 h at r.t.. Pentane was added until a white precipitate formed. Yield: 118 mg (71%); ^1^H NMR (300.13 MHz, CDCl_3_): *δ* = 8.31 (m, 1H), 7.66 (m, 1H), 7.51 (m, 11H), 6.96 (m, 1H) ppm; ^13^C{^1^H} NMR (75.47 MHz, CDCl_3_): *δ* = 168.55 (s), 134.85 (d, *J* = 7.24 Hz), 134.07 (d, *J* = 15.13 Hz), 133.11 (d, *J* = 8.18 Hz), 132.89 (d, *J* = 9.54 Hz), 132.83 (d, *J* = 7.08 Hz), 131.84 (s), 131.64 (d, *J* = 1.76 Hz), 130.42 (s), 129.57 (s), 129.19 (d, *J* = 12.13 Hz) ppm; ^31^P{^1^H} (121.49 MHz, CDCl_3_): *δ* = 35.97 (s) ppm; HRMS(ESI): *m/z* for [M + Na]^+^ calculated 561.0056, found 561.0094.

#### **Bromido-[2-(diphenylphosphino)benzoic acid]gold(I), (1-Br, C**_**19**_**H**_**15**_**AuBrO**_**2**_**P) 1-Cl**

 (17.6 mg, 32.7 µmol) was dissolved in 5 cm^3^ DCM. KBr (16.7 mg, 0.140 mmol) was dissolved in 5 cm^3^ water. After combining both solutions, the reaction mixture was stirred for 2 h at r.t. Phases were separated and the organic phase was dried over anhydrous Na_2_SO_4_. Evaporation of the solvent resulted a white solid. Yield: 17.4 mg (91%); ^1^H NMR (300.13 MHz, CDCl_3_): *δ* = 8.30 (m, 1H), 7.56 (t, ^*3*^*J*_*HH*_ = 7.56 Hz, 1H), 7.51 (m, 11H), 6.96 (m, 1H) ppm; ^13^C{^1^H} NMR (75.47 MHz, CDCl_3_): *δ* = 169.36 (s), 135.10 (d, *J* = 6.38 Hz), 134.15 (d, *J* = 14.27 Hz), 133.09 (s), 132.47 (s), 131.67 (s), 131.12 (s), 130.32 (s), 129.38 (d, *J* = 12.12 Hz) ppm; HRMS(ESI): *m/z* for [M + Na]^+^ calculated 604.9551, found 604.9593. Crystals suitable for single-crystal X-ray diffraction were obtained by slow gas-phase diffusion of pentane into a diluted DCM solution.

#### **Iodido-[2-(diphenylphosphino)benzoic acid]gold(I), (1-I, C**_**19**_**H**_**15**_**AuIO**_**2**_**P) 1-Cl**

 (10.0 mg, 0.02 mmol) was dissolved in 5 cm^3^ DCM. KI (20 mg, 0.12 mmol) was dissolved in 5 cm^3^ water. After combining both solutions, the reaction mixture was stirred for 2 h at r.t. Phases were separated and the organic phase was dried over anhydrous Na_2_SO_4_. Evaporation of the solvent resulted in a white solid. Yield: 10.8 mg (91%); ^1^H NMR (300.13 MHz, CDCl_3_): *δ* = 8.29 (m, 1H), 7.65 (t, ^*3*^*J*_*HH*_ = 7.73 Hz, 1H), 7.52 (m, 11H), 6.96 (m, 1H) ppm; ^13^C{^1^H} NMR (75.47 MHz, CDCl_3_): *δ* = 168.94 (s), 135.01 (d, *J* = 6.94 Hz), 134.17 (d, *J* = 15.13 Hz), 133.09 (s), 132.47 (s), 131.67 (s), 131.66 (s), 130.35 (s), 129.37 (d, *J* = 11.98 Hz) ppm; ^31^P{^1^H} NMR (121.49 MHz, CDCl_3_): *δ* = 42.5 (s) ppm; HRMS(ESI): *m/z* for [M + Na]^+^ calculated 652.9412, found 652.9468.

#### **Chlorido-tris[2-(diphenylphosphino)benzoic acid]gold(I), (2-Cl, C**_**57**_**H**_**45**_**AuClO**_**6**_**P**_**3**_**) 1-Cl**

 (118 mg, 0.219 mmol) was dissolved in dry DCM and cooled and 2-(diphenylphosphino)benzoic acid (134 mg, 0.437 mmol) was added to the solution and stirred for 2 h at r.t. The product directly precipitated from the reaction solution. Filtration of the product resulted in a white solid. The product was dried in vacuum. Yield: 217 mg (86%). Due to the low solubility of the compound, no NMR and MS spectra could be recorded. Crystals suitable for single-crystal XRD were obtained by addition of excess amounts of the respective ligand to a slurry of **2-Cl** in chloroform.

#### **Bromido-tris[2-(diphenylphosphino)benzoic acid]gold(I), (2-Br, C**_**57**_**H**_**45**_**AuBrO**_**6**_**P**_**3**_**) 1-Br**

 (98 mg, 0.17 mmol) was dissolved in chloroform and 2-(diphenylphosphino)benzoic acid (104 mg, 0.340 mmol) was added. After a few minutes of stirring at r.t. the product precipitated directly from the reaction solution. Excess solution was decanted and the white powder was washed with chloroform. The product was dried in vacuum. Yield: 191 mg (95%). Due to the low solubility of the compound, no NMR and MS spectra could be recorded.

#### **Iodido-tris[2-(diphenylphosphino)benzoic acid]gold(I), (2-I, C**_**57**_**H**_**45**_**AuIO**_**6**_**P**_**3**_**) 1-I**

 (95 mg, 0.15 mmol) was dissolved in chloroform and 2-(diphenylphosphino)benzoic acid (94 mg, 0.30 mmol) was added. After stirring for a few minutes, the product began to precipitate directly from the reaction solution. Excess solution was decanted and the white solid was washed with chloroform. The product was dried in vacuum. Yield: 175 mg (93%). Due to the low solubility of the compound, no NMR and MS spectra could be recorded.

## Supplementary Information

Below is the link to the electronic supplementary material.

Supplementary file1 (DOCX 314 KB)
