# Supplementary material for: A trigonal coordination of Au(I) phosphane complexes stabilized by O–H⋯X (X = Cl–, Br–, I–) interactions
Source: Monatsh Chem. 2021 Sep 15;152(10):1201–7. doi: 10.1007/s00706-021-02843-2 (PMC8550744; doi:10.1007/s00706-021-02843-2)
Supplement: Supplementary file 1 — Supplementary file1 (DOCX 314 KB) [file 706_2021_2843_MOESM1_ESM.docx]

Supporting Information

A trigonal coordination of Au(I) phosphine complexes stabilized by O–H^…^X (X = Cl^–^, Br^–^, I^–^) interactions

**Petra Gründlinger^1^ ● Cezarina Cela Mardare^2,3^ ● Thorsten Wagner^1^ ● Uwe Monkowius^4,^***

🖂 Uwe Monkowius

uwe.monkowius@jku.at

^1^ Institute of Experimental Physics – Surface Science Division, Johannes Kepler University Linz, Altenberger Straße 69, 4040 Linz, Austria

^2^ Institute of Chemical Technology of Inorganic Materials, Johannes Kepler University Linz, Altenberger Straße 69, 4040 Linz, Austria

^3^ Department of Physics and Chemistry of Materials, Faculty of Medicine/Dental Medicine, Danube Private University, Steiner Landstraße 124, 3500 Krems-Stein, Austria

^4^ School of Education, Chemistry, Johannes Kepler University Linz, Altenberger Straße 69, 4040 Linz, Austria


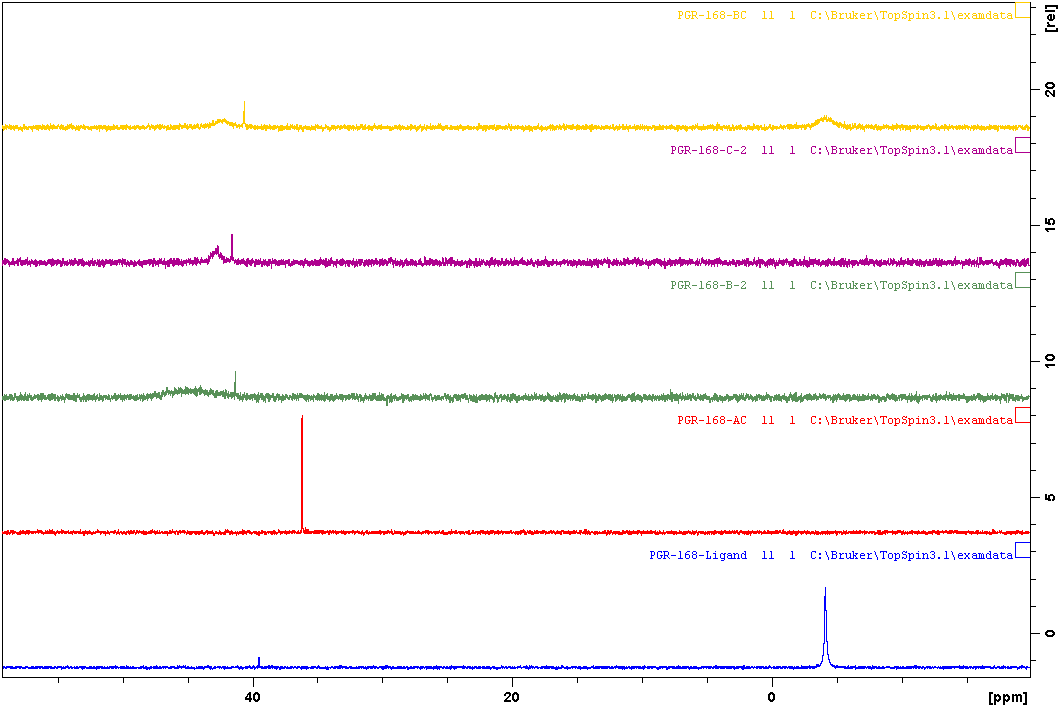


*

*

*

*

**c)**

**b)**

**a)**


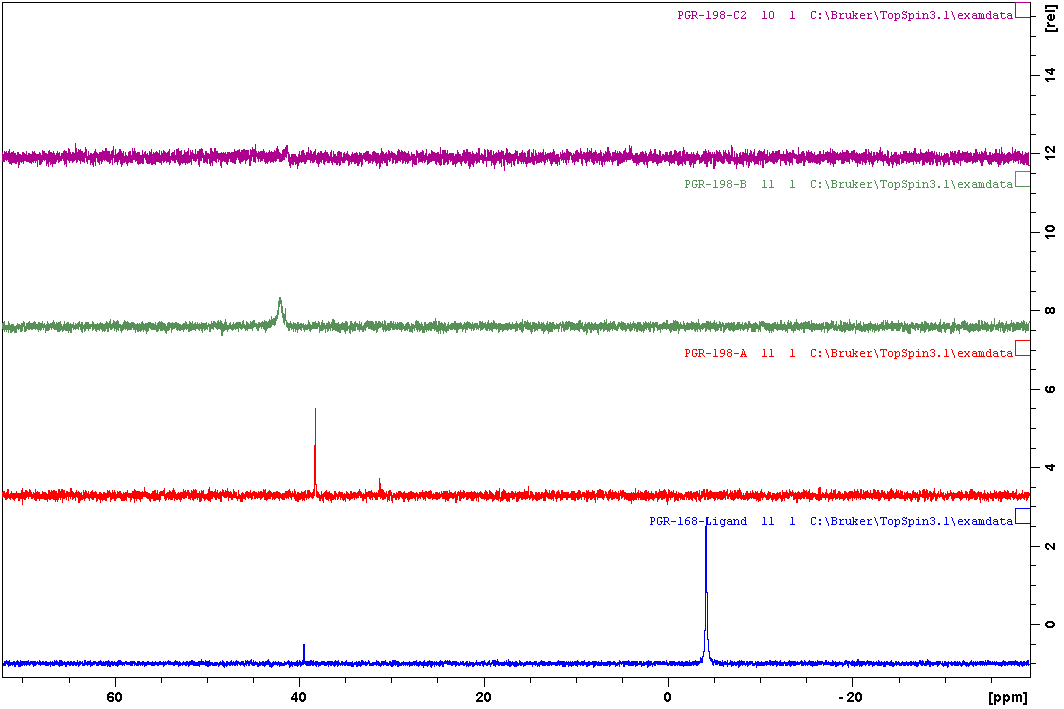

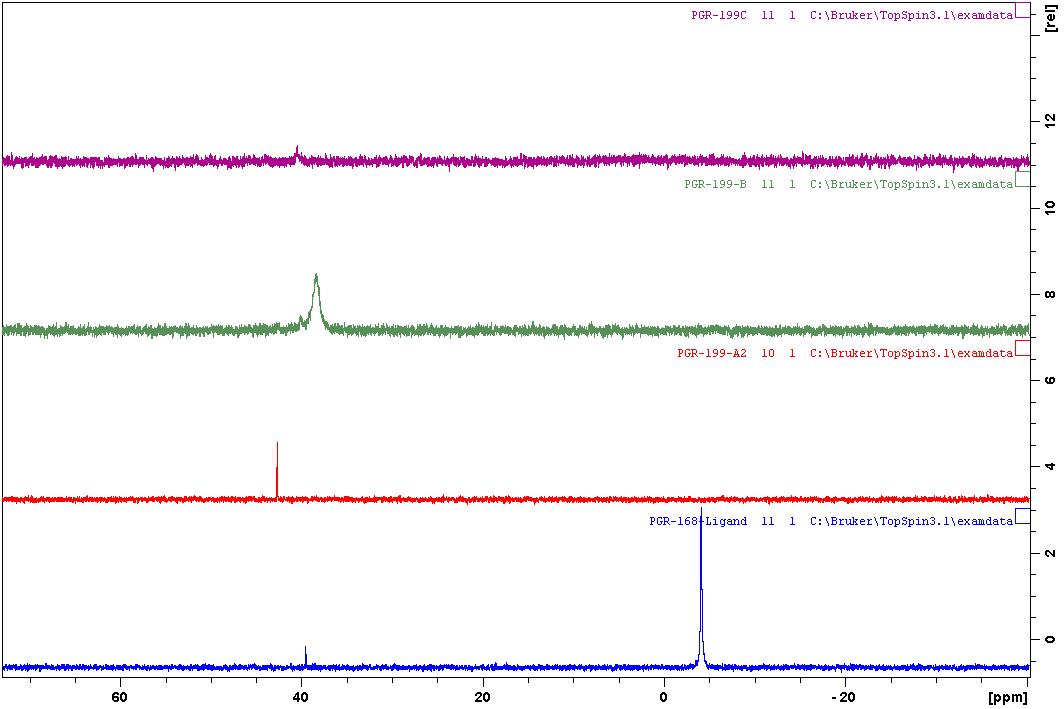


*

*

Figure S1: Comparison of ^31^P {^1^H} NMR spectra: a) from bottom to top: free ligand Ph_2_P(*o*-BzOH) (blue), 1-Cl (red), 1-Cl + 1 eq. ligand (green), 1-Cl + 2 eq. ligand (purple), 1-Cl + excess ligand (yellow). Same for 1-Br (b) and 1-I (c). (asterisk: phosphine oxide)

**Molecular and Crystal Structure of [Au(PPh_2_(*o*-BzOH))_2_I]**

**Iodido-bis[2-(diphenylphosphino)benzoic acid]gold(I), (3-I, C_38_H_30_AuIO_4_P_2_).** Some few crystals of **3-I** could be isolated in attempts to crystallize **2-I**. Unfortunately, we could not isolate pure **3-I** by addition of one equivalent 2-(diphenylphosphino)benzoic acid to **1-I**.

Crystals contain solvent molecules which could not be modelled reasonably. Their electron densities were removed by the SQUEEZE routine within PLATON. Further details can be found in the cif [^[[1]](#endnote-1)^].

The compound (Figure 3, top) crystallizes in the monoclinic space group I2/a. The asymmetric unit is composed of half of a complex molecule. The complex is thus *C*_2_-symmetric with the Au1–I1 vector as *C*_2_-axis. This leads to a trigonal-planar geometry around the gold atom with angles of 145.03° (P1–Au1–P1^i^) and 107.49° (P1–Au1–I1) with an angular sum of exact 360°. The distances Au1–P1 and Au1–I1 are 2.311 Å and 2.8519 Å, respectively. For comparison, in Ph_3_PAuI these distances are 2.2533(5) Å (Au–P) and 2.5633(3) Å (Au–I) [^[[2]](#endnote-2)^], which means that particularly the Au–I bond is considerably stretched. Furthermore, the carboxylic acid groups link the complexes to infinite one-dimensional zig-zag chains (see Fig. S2 bottom).


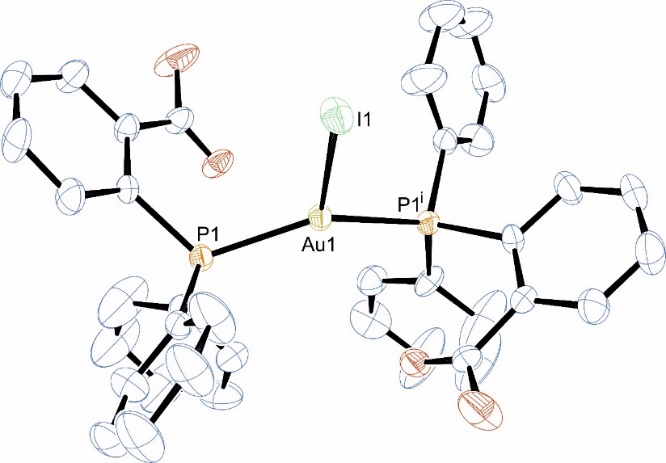


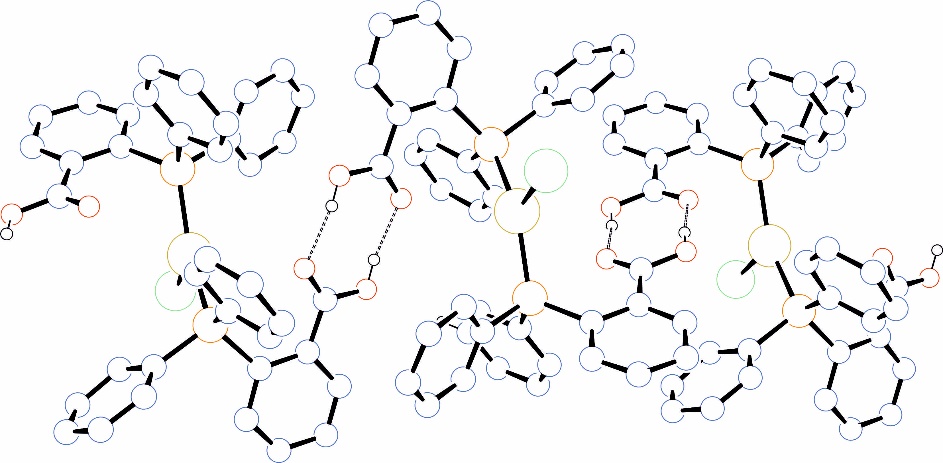


**Fig. 3** top: molecular structure of [Au(PPh_2_(*o*-BzOH))_2_I] (H omitted for clarity); bottom: excerpt from the cell plot illustrating the one-dimensional hydrogen bonded chain. Selected bond lengths in Å and angles in °: Au1–P1 2.311, Au1–I1 2.8519, P1–C1 1.846(9), P1–C8 1.828(6), P1–C14 1.809(6), P1–Au1–I1 107.49, P1–Au1–P1 145.03, C14–P1–Au1 115.5, C14–P1–C1 106.7(3), C14–P1–C8 101.1(3).

**Table S1.** Crystal data and data collection and structure refinement details for [Au(PPh_2_(*o*-BzOH))_2_I].

|  | [Au(PPh_2_(*o*-BzOH))_2_I] |
| --- | --- |
| Empirical Formula | C_38_H_30_AuIO_4_P_2_ |
| M_r_ / g mol^-1^ | 936.43 |
| Crystal Size / mm^3^ | 0.63×0.29×0.21 |
| Crystal System | monoclinic |
| Space group | *I*2/*a* |
| a / Å | 16.4373(5) |
| b / Å | 17.7767(5) |
| c / Å | 18.3429(8) |
| α / ° | 90 |
| β / ° | 110.996 |
| γ / ° | 90 |
| V / Å^3^ | 5003.95 |
| ρ_calcd._ / g cm^3^ | 1.243 |
| Z | 4 |
| μ(MoK_α_) / mm^-1^ | 3.65 |
| T / K | 296 |
| Θ Range / ° | 2.7-26.1 |
| Reflections collected | 89734 |
| Unique reflections | 5122 |
| Observed reflections I > 2σ(I) | 4060 |
| Absorption correction | multi-scan |
| T_min_/T_max_ | 0.21/0.51 |
| ∆ρ_fin_(max/min) / e Å^-3^ | 1.00, -1.50 |
| R1 (I ≥ 2σ(I)) | 0.049 |
| *w*R2 | 0.153 |
| CCDC | 2094497 |

1. . Spek AL (2015) Acta Crystallogr, Sect C: Cryst Struct Commun 71:9. [↑](#endnote-ref-1)
2. . Galassi R, Oumarou CS, Burini A, Dolmella A, Micozzi D, Vincenzetti S, Pucciarelli S (2015) Dalton Trans 44:3043 [↑](#endnote-ref-2)
